# The Leper Hospitals of the West Indies

**Published:** 1898-07-09

**Authors:** Louis Vintras

**Affiliations:** Physician to the French Hospital


### The Institutional Workshop.

### THE LEPER HOSPITALS OF THE WEST INDIES.

By Louis Vintras, M.D. Durh., B.Sc. Paris, Physician to the French Hospital.

II.

The same reservations are not to be made concerning the Trinidad Leper Asylum. Here we find most extensive grounds, situated some three miles from Port of Spain, and extending along a gentle slope almost to the sea shore. The asylum consists of separate pavilions, set well apart one from the other, each pavilion containing one ward, which runs through the whole length. The wards are high and well ventilated, clean and bright, but some are old, being, in fact, the old town barracks, and sadly deteriorated. These are gradually being pulled down and replaced by new buildings. There are 60 to 70 beds in some of the wards, which is certainly too many; but there is no bad smell, and it should be remembered that this asylum has to do duty for several islands, and the call made upon its accommodation is very great. The asylum is under the management of a small community of Catholic Sisters, who are most devoted to their work, and take the liveliest interest in their patients.

On arriving here and driving along the broad carriage-way, one loses entirely the sense of restraint which permeates most of these institutions. The patients are seen strolling about the grounds, or sitting on benches in the cool shade of the trees, or lying about on the grass, and certainly they have a much more contented appearance than in the other leper institutions which I have visited. Among all the unfortunate people I saw here I heard no complaint, while grumbling and discontent are generally freely expressed to visitors in the other institutions. Here the inmates take a lively interest in gardening, and most of the vegetables consumed in the asylum are grown in the grounds, the Government allowing the growers one-half the cost price, supplying them, of course, with both seeds and instruments. Other occupations are found for the women and also for some of the men, and are paid for on a similar scale; but more might still be done in this direction.

To the asylum is attached a medical superintendent, who, however, is non-resident. He visits the establishment frequently, and everything appertaining to the patients is under his immediate control. Dr. Knaggs, the present superintendent, has made several therapeutical essays in connection with this disease.

The water supply is not all that could be desired, but steps are being taken to remedy this defect.

One of the great difficulties in connection with the management of these institutions is the unruly spirit displayed by many of the inmates, and which seems to be a direct outcome of their morbid condition. The following, from one of the reports of Dr. Beaven Rake, a former medical superintendent of the Trinidad Leper Asylum, and whose reports on leprosy and on the work done in this institution are of the greatest interest, will give an idea of what has to be contended with in this direction.

"One man was sentenced at the criminal sessions to two years' imprisonment for seriously wounding another inmate with a cutlass, and three women were discharged for disorderly conduct. . . . There are certain chronic offenders, chiefly amongst the women, who persistently break the rules, and who are constantly being discharged and readmitted from year to year. This state of things seems unavoidable. Minor punishments are usually inflicted first, but as the offenders nearly always either refuse to submit to punishment or repeat the offence immediately afterwards, no alternative is left but discharge from the asylum This measure is no doubt deplorable on some grounds, but the importunity with which those discharged seek readmission shows that dismissal from the asylum is, as Gavin Milroy said it ought to be, the greatest punishment possible."

This last phrase is most significant, and I am able to vouch that in this institution, at least, dismissal is looked upon by the inmates as a punishment of which they show no small fear.

The asylum can accommodate about 220 lepers, and the following are the gross statistics for 1896: The number in the asylum on December 31st, 1895, was 209; there were 44 admissions during the year, 4 discharges, and 31 deaths.

The Leper Asylum of St. Kitts, one of the Leeward Islands, is well spoken of by all who have seen it. Unfortunately it is situated at Sandy Point, some distance from Basseterre, where the steamers call, and as they only stop a few hours it was impossible for me to visit the place. Dr. Foreman, the medical officer in charge, however, very kindly sent me the following particulars, for which I here beg to tender him my best thanks.

In 1894 "the institution was put under the management of the Poor Law Board for Sandy Point, resulting in a great saving to the Government. Of course everything that is of a professional character is under the immediate charge of the medical officer. A new master was also appointed, and gives general satisfaction. Several births having taken place in the asylum, although the sexes were divided by a double wooden fence, measures of a more severe kind had to be resorted to, so as to prevent the intercourse of the inmates. A barbed wire fence was erected lately, and up to the present has had the desired effect, notwithstanding the several attempts made by lepers to pass over it. During the year four lepers, one woman and three men, made their escape over the walls of the asylum for the purpose of visiting their friends. The police being informed of the fact as soon as those lepers were missed, they were invariably brought back to the institution the same day."

The punishment for this infringement of the rules consisted in being locked up for four days under a diet of bread and water every alternate day, and suppression of the tobacco and gin allowance for four days.

Dr. Foreman further comments on the latent discontent that exists among this class of patients, and adds: "What they (the lepers) feel mostly is, naturally, being deprived of their liberty, as, according to the regulations, they are never allowed to leave the asylum."

The admissions to the asylum for the last seven years were as follows: In 1891, 55 males and 33 females; in 1892, five males and two females; in 1893, four males and two females; in 1894, seven males and six females; in 1895, two males and two females; in 1896, two males and two females; in 1897, three males and one female; making a total of 126, while during the same period 58 died. There are some 30 lepers at large in the island, who, not being paupers, do not come under the Act.

The expenses for 1897 amounted to £1,047 8s. 4½d., being about 8½d. per head, including everything.

